# Musculoskeletal Injury and Physical Complaint Burden, Core Endurance, and Balance in Female Hip-Hop Dancers

**DOI:** 10.3390/sports14080327

**Published:** 2026-08-03

**Authors:** Onur Yüksel Öçal, Zeynep Meva Altaş, Mehmet Erdem Engin, Yunus Emre Meydanal, Cengizhan Özgürbüz

**Affiliations:** 1 Department of Sports Medicine, Ege University Faculty of Medicine, İzmir 35100, Türkiye; 2 Department of Sports Medicine, Hatay Training and Research Hospital, Hatay 31030, Türkiye; 3 Department of Public Health, Marmara University Faculty of Medicine, İstanbul 34854, Türkiye; 4 Department of Sports Medicine, Afyonkarahisar State Hospital, Afyonkarahisar 03030, Türkiye

**Keywords:** dancing, athletic injuries, injury surveillance, risk factors, postural balance

## Abstract

Hip-Hop dance is physically demanding, yet epidemiological data in female dancers remain limited. This mixed observational cohort study described musculoskeletal injury and physical complaint burden, evaluated core endurance and balance, compared female Hip-Hop dancers with healthy non-dancer controls, and examined associations with prospective complaints. Adult female Hip-Hop dancers (*n* = 31) and controls (*n* = 34) completed the McGill test battery, Unipedal Stance Test, and modified Star Excursion Balance Test. Dancers also reported prior-year injuries and musculoskeletal complaints and were followed for 12 weeks to record physical complaints and dance exposure. Dancers and controls showed similar anthropometric characteristics, core endurance, and balance scores (all *p* > 0.05). Musculoskeletal complaint incidence exceeded injury incidence during the previous year (46.1 vs. 0.72 per 1000 dance hours). During follow-up, 41.9% of dancers reported at least one physical complaint, corresponding to 36.6 physical complaints per 1000 dance hours. Trunk extensor endurance was inversely associated with physical complaint counts, whereas prior-year injury and musculoskeletal complaint counts were positively associated. Female Hip-Hop dancers demonstrated substantial overuse-related physical complaint burden despite relatively low injury incidence.

## 1. Introduction

Dance is both an art form and a physically demanding activity that requires strength, endurance, coordination, mobility, and motor control [1,2,3]. Because most dancers begin training at a young age and are exposed to repeated training loads, musculoskeletal problems are common in this community [2]. According to data cited by Ambegaonkar et al. [1] 50% to 85% of dancers are reported to sustain at least one dance-related musculoskeletal injury during a performance season, with overuse-related injuries and lower-extremity involvement being most common. However, the dance injury literature has focused predominantly on ballet, and relatively few studies have specifically examined Hip-Hop dancers [2,4].

Hip-Hop dance originated in New York in the 1970s and has since evolved into a broad movement domain that includes multiple substyles with distinct technical and physical demands [4]. Commonly described within “Old School” forms such as breaking, popping, and locking and “New School” forms such as house and krumping, Hip-Hop involves explosive transitions, floorwork, rapid directional changes, rhythmic isolations, and improvisational sequences [2,5,6]. These features place substantial demands on the musculoskeletal system and may increase injury risk through repetitive loading, impact, and multi-planar movement requirements [5,7].

Core stability has been proposed as one of the modifiable factors related to musculoskeletal injury risk [8]. The core is an integrated lumbopelvic system involving passive structures, active muscular components, and neural control mechanisms that together contribute to trunk stability, force transfer, and postural regulation [8,9]. Deficits in core function have been associated with lower extremity and low back injuries [8,10]. Repeated floor-to-standing transitions, rapid directional changes, landings, and multidirectional trunk movements require the lumbopelvic region to transfer force and maintain trunk control under repeated loading. These movement characteristics provide a biomechanical rationale for examining core stability and postural control in Hip-Hop dancers. Core endurance may therefore be relevant to fatigue resistance and movement control.

Balance is another clinically relevant factor in dance medicine because dancers must maintain postural control during single-leg tasks, landings, transitions, and rapid changes of direction. Hip-Hop dance also frequently requires single-leg support, rapid changes in the base of support, and control of the center of mass during dynamic transitions. Balance tests may provide feasible clinical indicators of postural-control capacity. Although better dynamic balance has been associated with lower injury risk in some dance populations [5,11], findings have been inconsistent and conventional balance tests may not fully reflect dance-specific sensorimotor demands [12,13].

Despite the physical demands of Hip-Hop dance, important gaps remain in the available literature. Dance-injury research has focused predominantly on ballet, while Hip-Hop dancers have been comparatively underrepresented. In addition, much of the Hip-Hop-specific epidemiological evidence has relied on retrospective injury reporting, and prospective studies combining physical performance assessment with complaint surveillance remain limited [2,4,5,6,7]. In the present study, physical complaint burden was operationally defined as the cumulative number of self-reported physical complaints recorded for each dancer during the 12-week follow-up. Examining feasible baseline measures of core endurance and balance together with prospective complaint monitoring may help determine whether these assessments have potential value for clinical follow-up and conditioning in Hip-Hop dancers. Therefore, the objectives of this study were to evaluate core endurance and static and dynamic balance performance in adult female Hip-Hop dancers, to compare these outcomes with those of healthy female non-dancer controls, to document injury and musculoskeletal complaint history over the previous year, and to prospectively monitor physical complaints over a 12-week period. We also aimed to compare dancers who did and did not report physical complaints during follow-up and to examine the relationships between physical complaint burden and performance test results. We hypothesized that dancers with better baseline core endurance and balance performance would report fewer physical complaints during follow-up, that dancers without physical complaints would perform better than those with physical complaints, and that Hip-Hop dancers would demonstrate better core endurance and balance performance than non-dancer controls.

## 2. Materials and Methods

### 2.1. Study Design

This study used a mixed observational design comprising cross-sectional and prospective components and was conducted at the Department of Sports Medicine, Ege University Faculty of Medicine, İzmir, Türkiye. The cross-sectional component compared female Hip-Hop dancers with healthy non-dancer controls in terms of core endurance and balance performance. In addition, dancers reported injuries and musculoskeletal complaints experienced during the previous year. The prospective component included a 12-week follow-up of the dancers only, during which physical complaints and dance exposure were recorded.

This study was approved by the Ege University Medical Research Ethics Committee (decision no. 24-5.1T/12; approval date: 23 May 2024) and was conducted in accordance with the Declaration of Helsinki. All participants provided written informed consent before enrollment. The study was registered at ClinicalTrials.gov (NCT06528652; first posted: 30 July 2024).

### 2.2. Participants and Sample Size

Between September 2024 and February 2025, 65 healthy female participants were included: 31 Hip-Hop dancers and 34 non-dancer controls. Eligible Hip-Hop dancers were recruited by invitation from local dance schools. The cohort was predominantly choreography-based and incorporated elements from multiple Hip-Hop substyles rather than representing a specific-style sample. Participants were not classified into mutually exclusive substyle categories, and formal professional or recreational status was not recorded. Healthy female non-dancer controls were recruited on a voluntary basis from university-affiliated staff, medical students, and the local community. Inclusion criteria were age 18 years or older, being free of musculoskeletal injury at baseline, and either competing in the adult category of Hip-Hop dance (dancer group) or not participating in dance (control group). Exclusion criteria were a history of spinal or abdominal surgery, pregnancy, use of medications that could affect balance, and any visual or balance disorder. Controls were not individually matched to dancers for age, BMI, or physical activity level. Physical activity outside dance was not quantified and was therefore not used as a matching or selection criterion. Their general physical activity level, participation in other sports, and resistance-training exposure were not assessed. Therefore, they were classified as non-dancer controls rather than inactive or sedentary controls.

Sample size was calculated using G*Power version 3.1.9.2. To detect a correlation of 0.50 between total injury count and balance/core endurance measures with α = 0.05 and 80% power, the required sample size was 29 dancers. A similar recruitment target was set for the control group. The study was completed with 65 participants.

### 2.3. Procedures

After written informed consent was obtained, anthropometric data were recorded, including height, weight, body mass index, leg length, and body fat percentage. Leg length was measured as the distance between the anterior superior iliac spine and the medial malleolus [14]. Lower-extremity dominance was determined by asking which foot the participant would use to kick a ball [14].

Dancers completed an investigator-developed baseline questionnaire prepared specifically for this study including age, Hip-Hop dance experience, weekly dance frequency and duration, and injury history during the previous year. The content domains and general question structure were informed by the studies reported by Ursej et al. [5] and Tjukov et al. [7], whereas the injury definitions and reporting framework were based on the IADMS recommendations [15,16]. The questionnaire was not a previously validated instrument and did not undergo separate validity or reliability testing.

**Figure 1:**
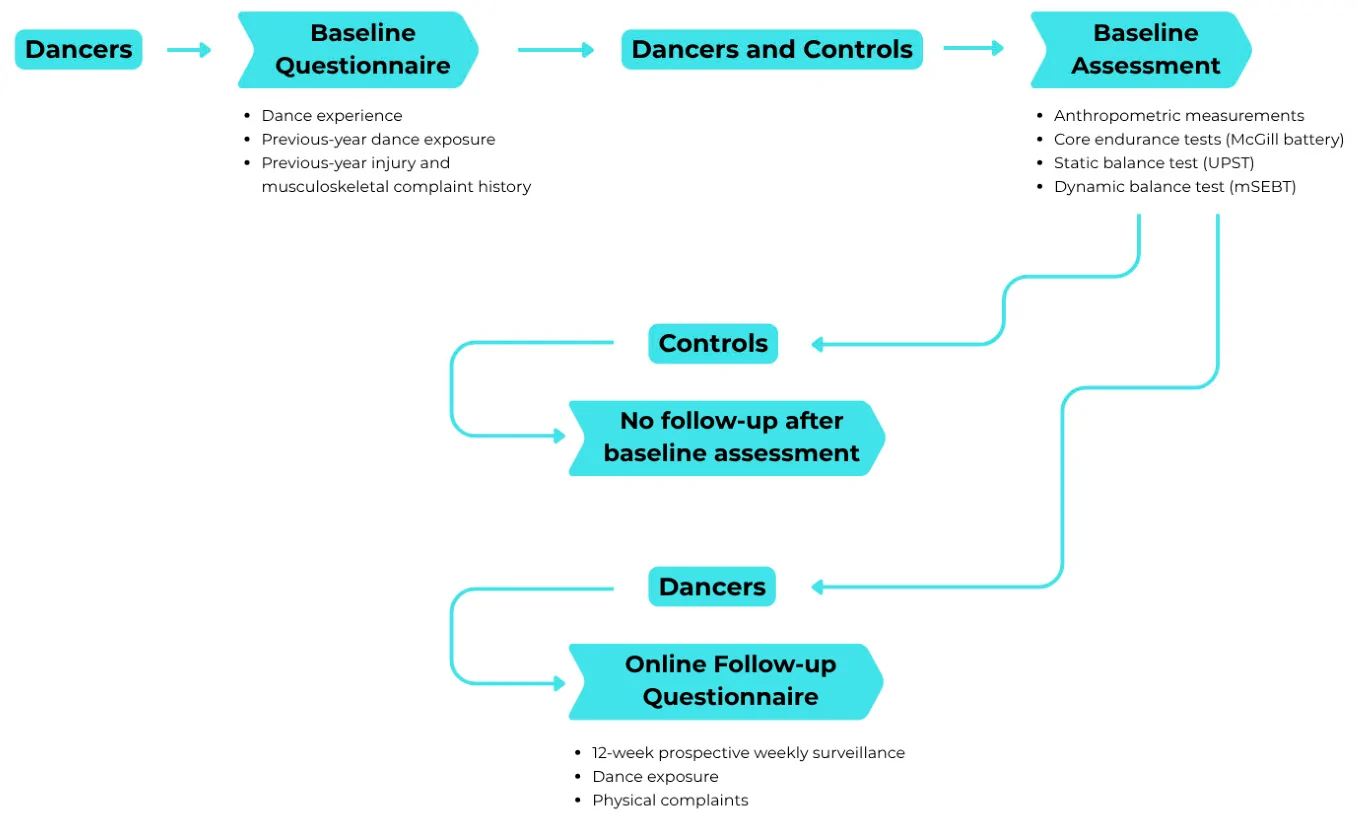
Study procedures and timeline. Arrows indicate the chronological sequence of procedures and the group-specific pathways. Both groups completed baseline anthropometric, core endurance, and balance assessments. Only dancers completed the retrospective baseline questionnaire and the subsequent 12-week weekly surveillance.

Participants received verbal and visual instructions for all performance tests. Before testing, each participant performed a self-selected 5 min warm-up. Core endurance tests were performed first, followed by static and dynamic balance testing, with a 2 min rest period between tests. Procedures are presented in Figure 1.

Core endurance was evaluated using the McGill test battery, which is widely used to assess core muscle endurance, and includes the trunk flexor endurance test, side bridge test, and trunk extensor endurance test [12,17,18]. Participants first performed a brief familiarization trial. The subsequent trial was measured in seconds, and the duration for which the correct test position could be maintained was documented. A 5 min rest period was provided between core endurance tests.

For the trunk flexor endurance test, participants were positioned with the hips and knees flexed to 90°, feet secured, arms crossed over the chest, and the trunk supported against a backrest angled at 60° to the examination table. After the support was removed, the duration for which the participant maintained the position was recorded [12,17,18]. The test was terminated if the trunk dropped below the target position, compensatory movements occurred, or fatigue was reported.

For the side bridge test, participants lay on one side with one foot placed in front of the other, hips and knees extended, and the elbow of the tested side positioned directly under the shoulder. The contralateral hand was placed across the chest on the tested-side shoulder. Participants were instructed to lift the pelvis and maintain the body in a straight line [17,18,19]. Test duration was recorded until alignment was lost, compensatory movements occurred, or fatigue or discomfort was reported. Both sides were tested, beginning with the dominant side.

For the Biering-Sorensen trunk extensor endurance test, participants lay prone with the anterior superior iliac spines aligned with the edge of the examination table. The lower body was secured with straps at the pelvis, knees, and ankles. Participants crossed their arms over the chest and maintained the upper body in a horizontal position off the table [17,18,19]. The duration was recorded until the upper trunk dropped below horizontal for more than 3 s, compensatory hyperextension occurred, or fatigue was reported.

Static balance was assessed using the Unipedal Stance Test (UPST) [20], and dynamic balance was assessed using the modified Star Excursion Balance Test (mSEBT) [14]. Both tests were performed bilaterally, beginning with the dominant side.

In the UPST, participants performed eyes-open and eyes-closed single-leg stance trials while barefoot. During eyes-open trials, they focused on a point at eye level on the wall. Participants stood on one leg with arms crossed over the chest and the non-stance foot held at the level of the medial malleolus. The test was terminated if the arms moved from the crossed position, the lifted foot touched the ground or moved away from the stance leg, the stance foot rotated or shifted, the eyes opened during eyes-closed trials, or 45 s was reached [20]. One test set including both eyes-open and eyes-closed conditions was repeated three times, with 5 min rest periods between sets. Best and mean values were recorded in seconds.

The mSEBT was performed using three tape lines arranged in an inverted Y configuration representing the anterior, posterolateral, and posteromedial directions [14,21,22]. Participants stood barefoot and, after verbal and visual instruction, completed three practice trials in each direction before formal testing [18,22]. During formal testing, three valid reaches were performed in each direction for both limbs. A 15 s rest period was provided between trials in the same direction and a 1 min rest period when changing limb or direction. Trials were repeated if the participant removed the hands from the hips, changed the stance-foot position, failed to return to the starting position, or transferred weight onto the reaching foot [18,22]. Maximum reach distance was normalized to leg length, and a composite score was calculated from the normalized reach distances in the three directions.

### 2.4. Injury and Complaint Definitions and Surveillance

For the retrospective assessment, injury was defined as a physician-diagnosed anatomical tissue impairment resulting in full absence from activity for at least one day beyond the day of onset [15]. Activity was defined as participation in a dance class, rehearsal, or performance [15]. A musculoskeletal complaint was defined as a physical problem that did not meet this injury definition [15].

Because many dance-related problems may not result in medical attention or time loss, physical complaint is used as the umbrella term for all musculoskeletal problems reported during the prospective surveillance, regardless of medical attention or time loss [16,23]. Physical complaint burden was operationally defined as the cumulative number of self-reported physical complaints recorded for each dancer during the 12-week follow-up and was used for calculation. Weekly surveillance was conducted for 12 weeks using a self-report electronic questionnaire. The weekly electronic surveillance questionnaire was also investigator-developed and followed the same general structure as the baseline questionnaire, but the recall period was limited to the preceding seven days. The questionnaire did not ask dancers to classify a complaint as new, ongoing, or recurrent. Therefore, reports from the same anatomical region in consecutive weeks could not be reliably linked as a single episode or distinguished as separate events. Dancers reported the activity context of the problem (class, rehearsal, performance/show), timing within the activity (beginning, middle, end), anatomical region (marked on a body diagram), diagnosis or treatment, perceived causes, and weekly dance exposure. Exposure was recorded as both total dance hours and total dance activities. Incidence per 1000 dance hours was calculated as (physical complaint burden/total dance hours) × 1000. Incidence per 1000 dance activities was calculated as (physical complaint burden/total dance activities) × 1000.

### 2.5. Statistical Analysis

Data were analyzed using Statistical Package for the Social Sciences (SPSS) version 24.0. Continuous variables were summarized as mean ± standard deviation or median (minimum-maximum), and categorical variables as number and percentage. Normality was assessed using the Shapiro–Wilk test. For the cross-sectional comparison between female Hip-Hop dancers and healthy non-dancer controls, group status was used as the independent grouping variable. The dependent variables included participant characteristics and physical performance measures: age, height, body mass, BMI, body fat percentage, trunk flexor endurance, right and left side bridge endurance, trunk extensor endurance, static balance outcomes measured by the Unipedal Stance Test, and dynamic balance outcomes measured by the modified Star Excursion Balance Test. Normally distributed continuous variables were compared between groups using the independent-samples Student’s *t*-test, whereas non-normally distributed continuous variables were compared using the Mann–Whitney U test. Spearman correlation analysis was performed in female Hip-Hop dancers to examine the associations between selected baseline and history-related variables (trunk extensor endurance, the number of prior-year injuries, and the number of prior-year musculoskeletal complaints) and prospective physical complaints recorded during the 12-week follow-up. Spearman’s rank correlation coefficients (ρ) are reported as effect-size estimates. Bias-corrected and accelerated (BCa) 95% confidence intervals were estimated using 5000 bootstrap resamples. Statistical significance was set at *p* < 0.05.

## 3. Results

Dancers and controls were comparable in age, anthropometric characteristics, core endurance, and primary static and dynamic balance measures (all *p* > 0.05; Table 1). Detailed UPST and mSEBT data are presented in Supplementary Table S1.

The dancers had a median Hip-Hop dance experience of 3.00 years (range, 0.08–20.00). During the previous year, they reported a median weekly dance duration of 6.00 h (range, 2.00–30.00) and a median of 3.00 dance activities per week (range, 2.00–20.00). During the 12-week prospective follow-up, the median cumulative exposure per dancer was 55.00 h (range, 11.00–239.00) and 35.00 dance activities (range, 7.00–234.00).

During the previous year, six dancers (19.4%) reported at least one injury, whereas 24 (77.4%) reported musculoskeletal complaints. Injury incidence was 0.72 per 1000 dance hours and 1.24 per 1000 dance activities, whereas musculoskeletal complaint incidence was 46.1 per 1000 dance hours and 79.4 per 1000 dance activities. Overuse was the predominant mechanism for both injuries and musculoskeletal complaints. The knee, shoulder, and neck were the most frequently affected regions for injuries, whereas musculoskeletal complaints most commonly involved the upper back, knee, thigh, and lower back. Most musculoskeletal complaints were managed without interruption of dance participation (Table 2).

Over the 12-week surveillance period, dancers accumulated 1997.5 dance hours across 1560 dance activities. Thirteen dancers (41.9%) reported at least one physical complaint, yielding 73 physical complaints overall. Incidence was 36.6 per 1000 dance hours and 46.8 per 1000 dance activities. Overuse was again the predominant mechanism, and the knee was the most commonly affected region (Table 3). Female Hip-Hop dancers with prospective physical complaints tended to be younger and had lower BMI and body fat percentage than those without prospective physical complaints; however, these differences were not statistically significant. Dancers with prospective physical complaints also had a shorter dance history, but their total dance exposure during the 12-week follow-up was higher. Regarding physical performance, core endurance times were shorter in dancers with prospective physical complaints, whereas balance test scores tended to be better. Nevertheless, none of these between-group differences reached statistical significance. Detailed results are presented in Supplementary Table S2.

Trunk flexor endurance, side bridge performance, and static and dynamic balance variables were not associated with prospective physical complaint counts (Supplementary Table S3). In contrast, trunk extensor endurance showed a significant inverse correlation with prospective physical complaint counts (r = −0.400, *p* = 0.026). Prior-year injury counts (r = 0.361, *p* = 0.046) and prior-year musculoskeletal complaint counts (r = 0.495, *p* = 0.005) were positively correlated with prospective physical complaint counts (Table 4).

## 4. Discussion

The principal findings of this study did not support our hypotheses that female Hip-Hop dancers would demonstrate superior core endurance and balance performance compared with non-dancer controls or that dancers without prospective physical complaints would perform better than those who reported physical complaints. Core endurance, static balance, and dynamic balance outcomes were generally comparable between groups, and baseline performance measures did not clearly distinguish dancers according to subsequent physical complaint status. Nevertheless, prospective surveillance demonstrated a substantial burden of predominantly overuse-related physical complaints, while prior-year injury and musculoskeletal complaint history were associated with greater prospective physical complaint burden. Among the baseline performance variables examined, only trunk extensor endurance showed a significant inverse association with prospective complaint count; this finding should be interpreted as exploratory.

Our retrospective injury incidence was lower than that reported in studies, including more breaking-focused Hip-Hop samples, but higher than injury incidence reported when breaking dancers were excluded. Ojofeitimi et al. [6] reported 2.37 injuries per dancer per year and 1.62 time-loss injuries per dancer per year in Hip-Hop dancers, with a higher burden in breakers and frequent involvement of the lower extremity, hand-wrist, shoulder, and neck regions. Tjukov et al. [7] reported an incidence of 1.156 per 1000 dance hours in Hip-Hop dancers overall, 2.456 per 1000 dance hours in breaking dancers, and 0.318 per 1000 dance hours when breaking was excluded. A systematic review also indicated that injuries in Hip-Hop and modern dancers are predominantly overuse-related and commonly affect the lower extremity [2]. The comparatively lower injury rate in our cohort may reflect differences in training profile, performance level, and style composition, as our participants were not recruited from a single sub-style and appeared to represent a more choreography-based training context.

Despite the relatively low rate of medically diagnosed time-loss injuries, musculoskeletal complaints were highly prevalent in our sample. This pattern is consistent with the idea that dancers may continue participating despite pain or discomfort and may not seek formal medical care for many problems. Tsiouti and Wyon reported that 44.5% of breaking dancers were already injured and that many managed injuries independently or continued dancing cautiously rather than seeking medical attention [24]. Similarly, Honrado et al. found that most presentations to on-site medical teams during breaking competitions were related to chronic rather than acute problems [25]. Taken together, these studies and our findings suggest that reliance on medically diagnosed or time-loss injuries alone may underestimate musculoskeletal burden in Hip-Hop dancers.

The knee was the most frequently affected region in both the retrospective and prospective data, consistent with previous reports in Hip-Hop dancers [4,5,7]. In our sample, the shoulder and neck were also commonly involved. This distribution aligns with the loading characteristics described in breaking and other Hip-Hop forms, in which lower-extremity demands coexist with substantial upper-extremity and cervical loading, particularly during floor-based and multidirectional movements [6,26,27].

A history of prior injury and musculoskeletal complaint was positively associated with the number of physical complaints reported during the 12-week follow-up. Although causality cannot be inferred, this finding is consistent with the broader dance injury literature. Swain et al. identified previous low back pain as an important risk factor in dancers [28], and Ursej et al. reported that a recent injury history increased the likelihood of subsequent injury in competitive Hip-Hop dancers [5]. Possible explanations include incomplete recovery, persistent neuromuscular deficits, altered movement patterns, and continued participation despite symptoms. From a clinical perspective, these findings support the value of monitoring recurrent or low-grade musculoskeletal complaints rather than focusing only on acute or time-loss injuries.

The high compliance achieved during prospective surveillance is an additional strength of this study. Dang et al. emphasized the utility of weekly online injury questionnaires in dance populations but also noted high dropout as a common limitation [29]. In our sample, compliance during the 12-week follow-up was 90.6%, with a median of 12 questionnaires completed per dancer, which strengthens confidence in the prospective findings.

With regard to McGill core endurance test performance, no significant between-group differences were found. Flexor, lateral, and extensor endurance values were generally comparable between dancers and controls, although extensor endurance tended to be lower in dancers. These values were similar to those reported in Latin dancers by Özkal et al. [30] but lower than values described in female elite athletes by Evans et al. [31]. Although trunk extensor endurance was numerically lower in dancers than in controls, this difference did not reach statistical significance and should therefore be interpreted cautiously. One possible explanation is that Hip-Hop training may emphasize repeated dynamic and multidirectional trunk actions more than sustained isometric trunk extension of the type assessed by the Biering–Sorensen test. However, physical activity and resistance-training exposure outside dance were not quantified in either group, and some control participants may have regularly engaged in activities that developed trunk endurance. The heterogeneity of the dancers’ training backgrounds and sampling variability associated with the modest sample size may also have contributed to this numerical difference. Consequently, the present findings cannot establish whether lower extensor endurance represents a dance-related adaptation or a true impairment.

Trunk extensor endurance within the dancer group was negatively associated with the total number of physical complaints during follow-up, whereas trunk flexor and side bridge performance were not. In addition, although core endurance did not differ significantly between dancers with and without physical complaints, trunk extensor endurance tended to be lower in those reporting physical complaints. However, several baseline variables were examined without formal adjustment for multiple comparisons, and this was the only significant association among the performance measures. The result should therefore be considered exploratory and may represent a chance finding. It does not establish that lower trunk extensor endurance causes physical complaints or that improving extensor endurance would reduce physical complaint risk. Replication in larger cohorts using pre-specified analyses and adjustment for relevant confounders is required. Prior studies link core-related deficits to musculoskeletal problems in dancers and athletes, although the implicated specific components vary across studies [10,19,32]. Given the lumbopelvic demands of Hip-Hop dance, including repeated flexion-extension, floor transitions, and abrupt postural changes, insufficient extensor endurance may plausibly contribute to reduced load tolerance. This hypothesis should be tested in larger prospective and interventional studies.

Static balance performance did not differ significantly between dancers and controls. Under eyes-open conditions, many participants in both groups reached the 45 s ceiling, limiting discrimination. Eyes-closed performance was also similar between groups. Previous studies have shown superior static balance in some dance populations [33,34], whereas another study has suggested that simple single-leg stance tests may be insufficiently sensitive to capture sensorimotor adaptations [35]. Our results are more consistent with the latter interpretation. Likewise, static balance was not associated with prospective physical complaint burden, suggesting that this measure may have limited value for identifying lower-severity physical problems in this context.

Similarly, dynamic balance assessed with the mSEBT did not distinguish dancers from controls and was not associated with physical complaint status during follow-up. Although previous studies have reported better dynamic balance in dancers than in non-dancers [22,36], these differences do not appear consistently across all populations or test formats [37]. Moreover, while some studies have linked SEBT performance to injury risk in athletes and dancers [5,11,38], another study has questioned its predictive value in dance populations [39]. As highlighted in the dance-specific assessment literature, standard balance tests may not fully reflect the movement demands of dance, and more challenging or dance-specific protocols such as the dsSEBT may be more informative [13,40].

The absence of between-group differences in balance performance may have several explanations. First, the marked ceiling effect during the eyes-open UPST limited the capacity of this test to discriminate between participants with relatively high balance ability. Second, the control group consisted of non-dancers rather than confirmed sedentary individuals, and some controls may have participated in sports or structured exercise that supported balance performance. Third, the dancer cohort was heterogeneous with respect to experience, training exposure, and Hip-Hop movement background, which may have increased within-group variability and obscured dance-related adaptations. Finally, although the mSEBT assesses dynamic postural control, it may not reproduce the floorwork, rapid transitions, rhythm-dependent movements, and multidirectional demands characteristic of Hip-Hop dance. These methodological explanations should be considered alongside the possibility that the groups genuinely had comparable balance capacities.

From a clinical and practical perspective, the findings suggest that monitoring in Hip-Hop dancers should not be limited to medically diagnosed or time-loss injuries. Regular brief surveillance of non-time-loss physical complaints may help identify persistent or recurrent problems that might otherwise remain unrecognized while dancers continue participating. Particular attention may be warranted for dancers with a recent history of injury or recurrent musculoskeletal complaints, as these variables were associated with greater complaint burden during follow-up. Trunk extensor endurance may be considered as one component of a broader physical assessment and conditioning program together with lower-limb strength, landing control, balance, and multidirectional neuromuscular training. Previous studies in broader athletic and healthy populations suggest that core training can improve dynamic balance [41], while limited evidence indicates potential benefits for dance and sport-skill performance [42]. Plyometric or unstable-surface activities may also influence dynamic balance and inter-limb asymmetry [43]. More specifically, a three-month core-stability program involving adolescent ballet and Hip-Hop dancers was associated with improvements in static core muscle performance, lumbar motor control, and low back pain [44]. However, these findings cannot be directly generalized to adult female Hip-Hop dancers, and none of these interventions can be considered preventive on the basis of the present study. The association observed in the present study does not establish causality and does not support a specific screening threshold or preventive intervention. Hip-Hop-specific randomized trials are required to determine whether targeted trunk and lower-limb conditioning reduces physical complaints or injury occurrence. In contrast, the UPST and mSEBT should not be used in isolation to estimate future physical complaint risk in female Hip-Hop dancers.

This study has several strengths, including the use of both retrospective and prospective data from the same cohort, high compliance with prospective surveillance, and calculation of burden per both dance hour and dance activity. The inclusion of a control group also allowed for the comparison of core endurance and balance performance. Several limitations should also be acknowledged. The sample size was modest, only female dancers were included, and substyle-specific analyses were not possible. The modest sample size limits the precision and stability of the estimated associations, as reflected by the width of the bootstrap confidence intervals; therefore, these effect-size estimates should be interpreted cautiously. Physical activity level and participation in sports or structured exercise outside dance were not quantified in either group; therefore, unmeasured differences in general activity level may have influenced the between-group comparisons. Core function was evaluated only through endurance-based measures, and the balance tests were not dance-specific. In addition, the 12-week follow-up period did not cover a full dance season, and no Turkish dance-specific injury-surveillance instrument with established validity and reliability was available at the time of data collection. Therefore, investigator-developed baseline and weekly questionnaires were used. Although their content was informed by previous studies and IADMS recommendations, the questionnaires did not distinguish new, ongoing, and recurrent physical complaints and did not undergo separate validity or reliability testing, which may have introduced measurement error and may limit comparability with other studies.

## 5. Conclusions

Female Hip-Hop dancers in this study demonstrated a substantial musculoskeletal burden characterized primarily by frequent physical complaints rather than by medically diagnosed time-loss injuries. Overuse was the dominant mechanism, the knee was the most commonly affected region, and prior injury and musculoskeletal complaint history were associated with greater physical complaint burden during follow-up. Although core endurance and balance performance did not differ significantly between dancers and controls, an exploratory inverse association was observed between trunk extensor endurance and prospective complaint count, although this finding requires confirmation. Conventional static and dynamic balance tests may have limited sensitivity for capturing the specific sensorimotor demands of Hip-Hop dance.

## Figures and Tables

**Table 1:** Baseline characteristics, core endurance, and primary balance measures in female Hip-Hop dancers and non-dancer controls.

Variable	Dancers (*n* = 31)	Controls (*n* = 34)	*p*-Value
Participant Characteristics			
Age, y	21.0 (18.0–44.0)	22.5 (18.0–37.0)	0.070
Height, cm	163.0 (152.0–179.0)	163.5 (153.0–178.5)	0.375
Body mass, kg	59.3 (44.6–101.3)	58.15 (43.3–95.9)	0.849
BMI, kg/m^2^	22.26 (17.24–38.6)	21.65 (16.6–33.98)	0.627
Body fat, %	24.5 ± 8.4	24.4 ± 7.2	0.978
Core Endurance			
Trunk flexor endurance, s	181.72 (35.46–481.07)	184.0 (61.01–610.81)	0.106
Right side bridge, s	57.91 ± 21.79	62.65 ± 31.03	0.482
Left side bridge, s	60.14 ± 22.13	63.87 ± 37.15	0.621
Trunk extensor endurance, s	127.35 ± 36.07	150.32 ± 61.29	0.068
Static Balance (UPST)			
RL, EO, mean, s	45.0 (28.82–45.00)	45.0 (25.74–45.00)	0.913
RL, EC, mean, s	16.99 (2.50–45.00)	17.92 (3.31–45.00)	0.386
LL, EO, mean, s	45.0 (30.75–45.00)	45.0 (16.47–45.00)	0.386
LL, EC, mean, s	17.94 ± 11.81	17.72 ± 10.62	0.937
Dynamic Balance (mSEBT)			
RL composite score, % leg length	93.41 (77.24–116.25)	90.31 (76.85–122.08)	0.813
LL composite score, % leg length	94.02 (81.71–118.33)	91.02 (75.93–118.26)	0.958

Note. Values are presented as mean ± standard deviation or median (minimum-maximum), as appropriate. RL = right leg; LL = left leg; EO = eyes open; EC = eyes closed; UPST = Unipedal Stance Test; mSEBT = modified Star Excursion Balance Test.

**Table 2:** Retrospective injury and musculoskeletal complaint characteristics during the previous year in female Hip-Hop dancers.

Variable	Injury	MSK Complaint
Dancers reporting at least 1 episode, *n* (%)	6 (19.4)	24 (77.4)
Total number of episodes	9	578
Incidence per 1000 dance hours	0.72	46.1
Incidence per 1000 dance activities	1.24	79.4
Predominant mechanism	Overuse	Overuse
Most frequently affected region(s)	Knee, shoulder, neck	Upper back, knee, thigh, lower back
Stopped dancing due to symptoms, *n*	6	4
Continued dancing despite symptoms, *n*	NA	20
Median time-loss, days (minimum-maximum)	7 (2–90)	3.5 (2–7)

Note. Injury was defined as a physician-diagnosed episode causing full time-loss from activity for at least one day beyond onset. MSK = musculoskeletal, NA = not applicable.

**Table 3:** Prospective physical complaints during the 12-week surveillance period in female Hip-Hop dancers.

Variable	Value
Dancers with at least 1 physical complaint, *n* (%)	13 (41.9)
Total number of physical complaints	73
Total dance hours	1997.5
Total dance activities	1560
Incidence per 1000 dance hours	36.6
Incidence per 1000 dance activities	46.8
Mean physical complaints per dancer	2.35
Mean physical complaints per symptomatic dancer	5.6
Median physical complaints per dancer	0 (0–32)
Median physical complaints per symptomatic dancer	2 (1–32)
Predominant mechanism	Overuse
Most frequently affected region(s)	Knee, foot-ankle, neck, lower leg
Stopped dancing due to symptoms, *n*	5
Median time-loss, days (minimum-maximum)	1 (1–6)

Note. Prospective surveillance covered 12 weeks using weekly self-report questionnaires.

**Table 4:** Correlations between baseline variables, prior injury and musculoskeletal complaint counts with prospective physical complaint counts during the 12-week follow-up.

Variables Correlated with Prospective Physical Complaint Counts	Spearman ρ	BCa 95% CI	*p*-Value
Trunk extensor endurance	−0.400	−0.682 to −0.016	0.026
Number of prior-year injuries	0.361	−0.063 to 0.684	0.046
Number of prior-year musculoskeletal complaints	0.495	0.133 to 0.762	0.005

Note. Spearman’s rank correlation coefficients (ρ) are presented as effect-size estimates. Bias-corrected and accelerated (BCa) 95% confidence intervals were calculated using 5000 bootstrap resamples. BCa = bias-corrected and accelerated; CI = confidence interval.

## Data Availability

The datasets generated and/or analyzed during the current study are not publicly available due to participant confidentiality and institutional data protection considerations but are available from the corresponding author on reasonable request.

## Supplementary Materials

The following supporting information can be downloaded at https://www.mdpi.com/article/10.3390/sports14080327/s1. Table S1: Detailed static and dynamic balance data in female Hip-Hop dancers and non-dancer controls; Table S2: Comparisons between female Hip-Hop dancers with and without prospective physical complaints; Table S3: Non-significant correlations between baseline performance variables and prospective physical complaint counts in female Hip-Hop dancers. Supplementary Tables S1–S3 are provided as Supplementary Materials.

## Abbreviations

The following abbreviations are used in this manuscript:

BCa	Bias-corrected and accelerated
BMI	Body mass index
CI	Confidence interval
dsSEBT	Dance-specific Star Excursion Balance Test
mSEBT	Modified Star Excursion Balance Test
MSK	Musculoskeletal
SPSS	Statistical Package for the Social Sciences
UPST	Unipedal Stance Test
