# Supplementary material for: Musculoskeletal Injury and Physical Complaint Burden, Core Endurance, and Balance in Female Hip-Hop Dancers
Source: Sports (Basel). 2026 Aug 3;14(8):327. doi: 10.3390/sports14080327 (PMC13517429; doi:10.3390/sports14080327)
Supplement: Supplementary file 1 [file sports-14-00327-s001.zip › Supplementary Tables.pdf]

Supplementary Table S1. Detailed static and dynamic balance data in female Hip-Hop dancers and non-dancer controls

Variable	Dancers (n = 31)	Controls (n = 34)	p-value
**Static balance (UPST)**			
RL, EO, best, s	45.0 (45.0-45.0)	45.0 (39.29-45.0)	0.340
RL, EO, mean, s	45.0 (28.82-45.00)	45.0 (25.74-45.00)	0.913
RL, EC, best, s	24.98 (2.91-45.00)	36.37 (6.01-45.00)	0.326
RL, EC, mean, s	16.99 (2.50-45.00)	17.92 (3.31-45.00)	0.386
LL, EO, best, s	45.00 (45.00-45.00)	45.00 (22.11-45.00)	0.340
LL, EO, mean, s	45.0 (30.75-45.00)	45.0 (16.47-45.00)	0.386
LL, EC, best, s	27.79 (3.41-45.00)	24.07 (3.66-45.00)	0.894
LL, EC, mean, s	17.94 ± 11.81	17.72 ± 10.62	0.937
**Dynamic balance (mSEBT, normalised reach distances)**			
RL, ANT, % leg length	77.77 ± 5.26	79.24 ± 8.29	0.393
RL, PL, % leg length	101.84 ± 11.64	101.35 ± 12.07	0.870
RL, PM, % leg length	101.02 ± 13.08	100.69 ± 12.86	0.917
RL, composite score, % leg length	93.41 (77.24-116.25)	90.31 (76.85-122.08)	0.813
LL, ANT, % leg length	78.85 (69.51-91.95)	77.07 (65.85-103.26)	0.953
LL, PL, % leg length	101.28 (81.40-135.00)	101.20 (83.70-132.48)	0.788
LL, PM, % leg length	98.78 (78.16-133.75)	95.03 (70.00-129.94)	0.768
LL, composite score, % leg length	94.02 (81.71-118.33)	91.02 (75.93-118.26)	0.958

Note. Values are presented as mean ± standard deviation or median (minimum-maximum), as appropriate. RL = right leg; LL = left leg; EO = eyes open; EC = eyes closed; ANT = anterior; PL = posterolateral; PM = posteromedial; UPST = Unipedal Stance Test; mSEBT = modified Star Excursion Balance Test.

Supplementary Table S2. Comparisons between female Hip-Hop dancers with and without prospective physical complaints

Variable	Complaint absent (n = 18)	Complaint present (n = 13)	p-value
**Participant characteristics**			
Age, y	22.50 (18.00-44.00)	21.00 (18.00-26.00)	0.196
BMI, kg/m^2	22.88 (17.24-30.34)	21.53 (18.79-38.60)	0.548
Body fat, %	26.15 (8.90-41.10)	22.40 (14.30-44.70)	0.841
**Dance exposure/history**			
Dance experience, y	3.67 (0.08-20.00)	3.00 (0.08-7.00)	0.587
Weekly dance hours during previous year	6.00 (2.00-13.00)	4.00 (3.00-30.00)	0.761
Weekly dance activities during previous year	3.00 (2.00-10.00)	4.00 (2.00-20.00)	0.325
Total dance hours during 12-week surveillance	54.50 (11.00-125.00)	61.00 (23.50-239.00)	0.904
Total dance activities during 12-week surveillance	35.00 (7.00-89.00)	34.00 (13.00-234.00)	0.826
**Core endurance**			
Trunk flexor endurance, s	181.92 (53.13-394.27)	176.57 (35.46-481.07)	0.522
Right side bridge, s	64.54 (26.54-87.31)	45.61 (11.54-100.13)	0.689
Left side bridge, s	61.67 (7.99-88.96)	54.04 (14.33-110.50)	0.810
Trunk extensor endurance, s	123.56 (93.76-205.00)	114.34 (41.44-163.99)	0.066
**Balance**			
RL UPST, EC, mean, s	13.99 (2.50-35.82)	16.99 (5.30-45.00)	0.150
LL UPST, EC, mean, s	12.61 (3.15-42.08)	17.45 (6.19-40.11)	0.161
RL mSEBT composite score, % leg length	93.12 (77.24-116.25)	93.72 (77.78-106.62)	0.968
LL mSEBT composite score, % leg length	91.06 (81.71-118.33)	94.35 (83.86-106.59)	0.603

Note. Values are presented as mean ± standard deviation or median (minimum-maximum), as appropriate. RL = right leg; LL = left leg; EC = eyes closed; UPST = Unipedal Stance Test; mSEBT = modified Star Excursion Balance Test.

Supplementary Table S3. Non-significant correlations between baseline performance variables and prospective physical complaint counts in female Hip-Hop dancers

Variable	r	p-value
Trunk flexor endurance	-0.220	0.234
Right side bridge	-0.146	0.433
Left side bridge	-0.112	0.549
RL UPST, EO, mean	0.060	0.747
RL UPST, EC, mean	0.229	0.214
LL UPST, EO, mean	-0.103	0.580
LL UPST, EC, mean	0.184	0.323
RL mSEBT composite score, % leg length	0.050	0.788
LL mSEBT composite score, % leg length	0.137	0.462

Note. RL = right leg; LL = left leg; EO = eyes open; EC = eyes closed; UPST = Unipedal Stance Test; mSEBT = modified Star Excursion Balance Test.
